# Cord blood proteomics identifies biomarkers of early-onset neonatal sepsis

**DOI:** 10.1172/jci.insight.193826

**Published:** 2025-06-10

**Authors:** Leena B. Mithal, Mark E. Becker, Ted Ling-Hu, Young Ah Goo, Sebastian Otero, Aspen Kremer, Surya Pandey, Nicola Lancki, Yawei Li, Yuan Luo, William Grobman, Denise Scholtens, Karen K. Mestan, Patrick C. Seed, Judd F. Hultquist

**Affiliations:** 1 Department of Pediatrics, Division of Infectious Diseases, Ann & Robert H. Lurie Children’s Hospital of Chicago, Feinberg School of Medicine, Northwestern University, Chicago, Illinois, USA.; 2 Stanley Manne Children’s Research Institute, Ann & Robert H. Lurie Children’s Hospital of Chicago, Chicago, Illinois, USA.; 3 Division of Infectious Diseases, Departments of Medicine and Microbiology-Immunology, and; 4 Center for Pathogen Genomics and Microbial Evolution, Havey Institute for Global Health, Feinberg School of Medicine, Northwestern University, Chicago, Illinois, USA.; 5 Mass Spectrometry Technology Access Center at McDonnell Genome Institute (MTAC@MGI), Washington University School of Medicine, St. Louis, Missouri, USA.; 6 Department of Pediatrics, Division of Infectious Diseases, University of Chicago, Chicago, Illinois, USA.; 7 Department of Medicine, Division of Hematology and Oncology, and; 8 Department of Preventive Medicine, Division of Biostatistics and Informatics, Feinberg School of Medicine, Northwestern University, Chicago, Illinois, USA.; 9 Department of Obstetrics and Gynecology, Brown University, Providence, Illinois, USA.; 10 Department of Pediatrics, Division of Neonatology, UCSD, La Jolla, California, USA.

**Keywords:** Immunology, Infectious disease, Bacterial infections, Biomarkers, Proteomics

## Abstract

**BACKGROUND:**

Symptoms of early-onset neonatal sepsis (EOS) in preterm infants are nonspecific and overlap with normal postnatal physiological adaptations and noninfectious pathologies. This clinical uncertainty and the lack of reliable EOS diagnostics results in liberal use of antibiotics in the first days to weeks of life, leading to increased risk of antibiotic-related morbidities in infants who do not have an invasive infection.

**METHODS:**

To identify potential biomarkers for EOS in newborn infants, we used unlabeled tandem mass spectrometry proteomics to identify differentially abundant proteins in the umbilical cord blood of infants with and without culture-confirmed EOS. Proteins were then confirmed using immunoassay, and logistic regression and random forest models were built, including both biomarker concentration and clinical variables to predict EOS.

**RESULTS:**

These data identified 5 proteins that were significantly upregulated in infants with EOS, 3 of which (serum amyloid A, C-reactive protein, and lipopolysaccharide-binding protein) were confirmed using a quantitative immunoassay. The random forest classifier for EOS was applied to a cohort of infants with culture-negative presumed sepsis. Most infants with presumed sepsis were classified as resembling infants in the control group, with low EOS biomarker concentrations.

**CONCLUSION:**

These results suggest that cord blood biomarker screening may be useful for early stratification of EOS risk among neonates, improving targeted, evidence-based use of antibiotics early in life.

**FUNDING:**

NIH, Gerber Foundation, Friends of Prentice, Thrasher Research Fund, Ann & Robert H. Lurie Children’s Hospital, and Stanley Manne Children’s Research Institute of Lurie Children’s.

## Abstract 2

A diagnostic model of umbilical cord blood biomarker proteins identified by mass spectrometry proteomics distinguishes infants with early-onset neonatal sepsis from control infants.

## Graphical abstract

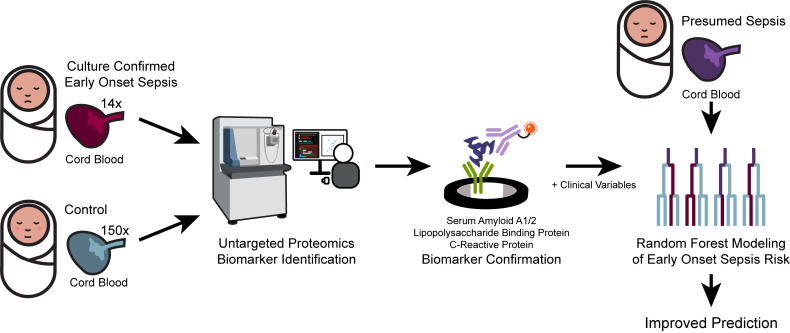

## Introduction

Early-onset neonatal sepsis (EOS) results in significant morbidity and mortality that disproportionately affects preterm infants (1–5). Clinical management of EOS is complicated by underlying uncertainty in the diagnosis due to undifferentiated symptoms, common risk factors, and lack of timely, reliable diagnostics (6–8). This uncertainty drives early antibiotic use in infants with any suspicion of sepsis. Antibiotics are the most prescribed medication in neonatal intensive care units, with substantial variation by practice, and most antibiotic exposures occur in infants who have negative bacterial cultures (i.e., presumed, culture-negative sepsis) (9, 10). In addition to growing antimicrobial resistance, neonates exposed to antibiotics without proven infection face increased near-term risks of fungal infection, late-onset sepsis, necrotizing enterocolitis, and death (11, 12). Early antibiotic exposure may also cause long-term morbidity, driven by resultant microbiome alterations (dysbiosis) and attendant immunologic and metabolic dysregulation (13, 14). More accurate and timely diagnostics are needed to identify or rule out EOS in neonates to reduce the burden of antibiotic use in this vulnerable population.

Several approaches to improve the rapid, accurate diagnosis of EOS have been devised (15, 16). Molecular pathogen diagnostics and placental pathology are valuable tools; however, they often suffer from delayed time to results, low positive predictive value, and limited availability (17). Laboratory markers of infection, including hematologic indices and markers of inflammation, such as C-reactive protein (CRP) and procalcitonin (18), have poor specificity and can be elevated due to non-infectious causes early in life, potentially leading to overtreatment with antibiotics (19, 20). Vital signs and heart rate variability monitoring algorithms have shown some benefits in triggering evaluations for late-onset sepsis occurring after the first 72 hours of life but not for EOS (21, 22). More holistic tools for risk assessment have also been developed. For example, the Kaiser Permanente Sepsis calculator accounts for various clinical risk factors, including intrapartum antibiotic prophylaxis, duration of rupture of membranes, gestational age (GA), and clinical status. This tool has reduced antibiotic exposure for newborns but can only be applied to infants greater than 34 weeks GA at birth, excluding low-birth-weight or infants who were born earlier who are at highest risk of EOS, EOS-related morbidity and mortality, and from harms of antibiotic overuse (23).

Several studies have investigated specific markers in umbilical cord blood that might improve the diagnosis of EOS. In most cases of EOS, an ascending infection affects the placenta, amniotic fluid, and fetus prior to birth, especially in the setting of spontaneous preterm labor. Umbilical cord blood reflects the intrauterine environment where EOS is seeded and the infant’s state at birth, unaffected by postnatal stressors and physiology. This, along with the ready availability of cord blood, makes it an attractive target for diagnostics. Previous studies by our group have demonstrated elevated levels of CRP, serum amyloid A (SAA), and haptoglobin in culture-proven EOS and in a small subset of presumed sepsis (PS) cases (24). Premature expression of haptoglobin in cord blood was similarly associated with EOS by another group (25). Other studies have implicated potential biomarkers, including presepsin (a soluble CD14 fragment released by myeloid cells during an immunological response) and CD64 (26–28). However, this research was limited to investigating a priori*–*selected markers and pooled analysis of culture-proven EOS and PS specimens.

Our objective was to use unbiased mass spectrometry proteomics to identify proteins that were differentially abundant in cord blood of infants with EOS compared with infants without EOS. After immunoassay validation, these data were used to develop a machine learning diagnostic model incorporating cord blood biomarkers and clinical factors to accurately identify and rule out EOS in newborn infants.

## Results

### Identification of EOS biomarkers in cord blood.

Table 1 displays the characteristics of the cohort across sepsis groups. Among the EOS cohort, known risk factors for EOS development were overrepresented. Infants with EOS had lower GAs as compared with infants in the control cohort (mean, 30.7 ± 3.3 weeks vs. 33.6 ± 4.5 weeks; 0% >37 weeks, 0% EOS vs. 27% controls) and were less frequently female (36% female vs. 49% female). Chorioamnionitis (43% vs. 0.7%), prolonged rupture of membranes (PROM) (64% vs. 11.3%), and vaginal delivery (71% vs. 51%) were likewise more common in patients with EOS compared with individuals acting as controls. The PS cohort resembled the EOS cohort with a greater frequency of prematurity, chorioamnionitis, and PROM than in the control group. Confirmed pathogens in blood culture included *E. coli* (*n* = 8), *Streptococcus agalactiae* (*n* = 2), *Klebsiella oxytoca* (*n* = 1)*, Proteus mirabilis* (*n* = 1)*, Haemophilus influenzae* (*n* = 1)*,* and *Listeria monocytogenes* (*n* = 1).

**Figure 1:**
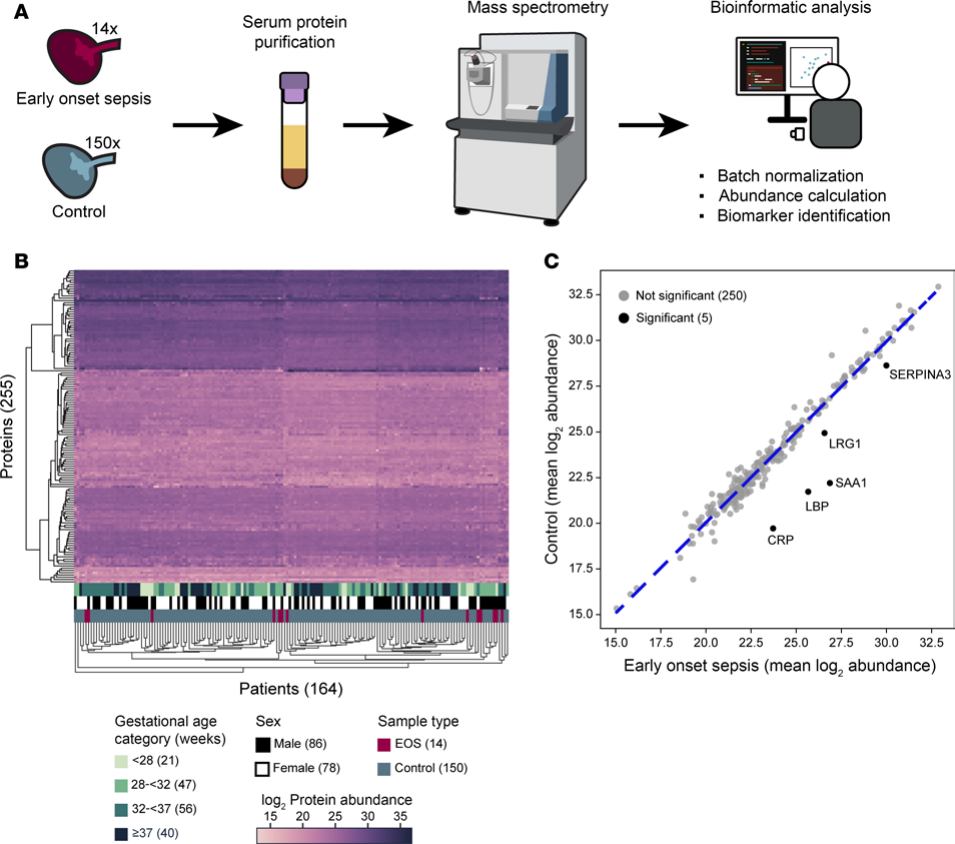
Proteomic identification of differentially abundant proteins in early-onset sepsis cord blood. (**A**) Diagram of workflow. (**B**) Hierarchically clustered heatmap of protein abundance in cord blood for EOS and control specimens. Specimens are clustered by gestational age category, sex, and sample type. Missing values were imputed. (**C**) Plot of mean abundance of proteins in EOS and control specimens. Black points were significant by Mann-Whitney *U* test with Benjamini-Hochberg FDR adjustment (*P* < 0.05).

To assess protein abundance, we performed liquid chromatography with tandem mass spectrometry (LC-MS/MS) on sera isolated from frozen, banked blood specimens from our control and EOS cohorts (Figure 1A). Spectra were analyzed using MaxQuant for label-free quantification (LFQ) of protein abundance (29). Peptides corresponding to 437 proteins were detected. After batch normalization, we visualized the protein abundance of the 255 most commonly detected proteins (present in >20% of specimens) across all 164 specimens using a hierarchically clustered heatmap. For heatmap visualization, missing values were imputed separately for EOS and control specimens either by iterative imputation for proteins in >70% or by sampling from the lowest decile of protein abundance for proteins in <70% of specimens. When visualized using hierarchical clustering, there were no clear changes in the plasma proteome that clustered by GA, sex, or specimen type (i.e., EOS or control) (Figure 1B). Clustering with naive imputation of zeros for all missing values revealed similar results (Supplemental Figure 1; supplemental material available online with this article; https://doi.org/10.1172/jci.insight.193826DS1).

These data suggest that any changes in the plasma proteome during EOS would be driven by a small number of proteins as opposed to global restructuring. To assess per protein changes in abundance, we plotted the mean abundance of each of the 255 most commonly detected proteins in our control specimens versus our EOS specimens (Figure 1C). Five proteins were significantly enriched in the plasma of infants with culture-confirmed EOS: CRP, lipopolysaccharide-binding protein (LBP), SAA1, leucine-rich α-2-glycoprotein 1 (LRG1), and serine proteinase inhibitor A3 (SERPINA3) (Figure 1C). These are all acute-phase reactant proteins that are upregulated in the plasma in response to inflammation. A sensitivity analysis was performed repeating differential abundance analysis between EOS specimens and GA-matched control specimens (excluding the full-term group, ≥37 weeks gestation). Results were consistent, with the same 5 proteins significantly elevated in the EOS specimens (Supplemental Figure 2A). Due to the small number of female infants with EOS (*n* = 4), statistical power was not adequate to detect differences of plausible magnitude for each sex.

As there were many fewer EOS specimens compared with control specimens, proteins found exclusively or predominantly in EOS cord blood could be excluded by our removal of proteins present in <20% of samples (*n* = 182 proteins). As these proteins might be superior predictors of EOS, we also assessed differential presence and absence of these less frequently detected proteins. Of the 182 proteins detected in fewer than 20% of specimens, only 3 were more often found in EOS specimens: SAA2, which was observed in 71% of EOS specimens but 2% of control specimens; haptoglobin/haptoglobin-related protein, which was observed in 43% of EOS specimens but 17% of control specimens; and lipocalin-2 (LCN2), which was observed in 36% of EOS specimens but 9% of control specimens. Notably, these proteins are also all acute-phase reactants, with SAA2 sequence and function largely redundant with SAA1. Given their rarity in the control specimens, we could not statistically compare their abundance across cohorts, so we focused on the 5 acute-phase reactants identified above.

### EOS cord blood samples contain higher levels of acute-phase reactant proteins.

**Figure 2:**
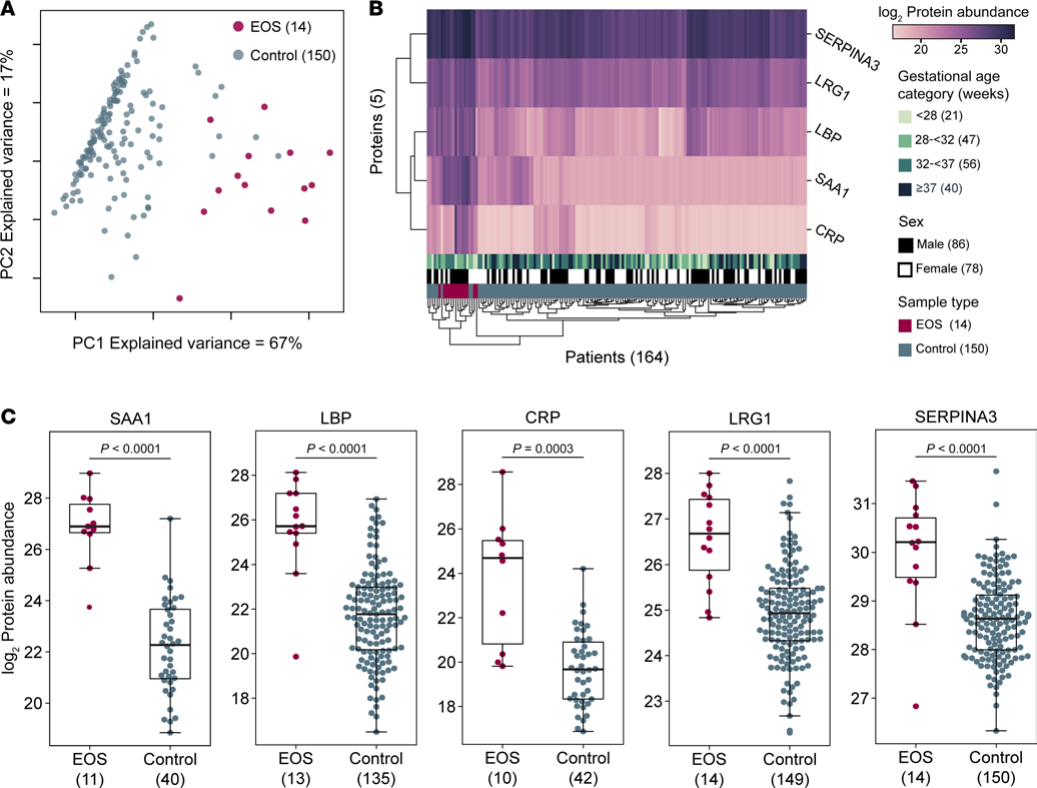
Details of differentially abundant proteins. (**A**) PCA and (**B**) clustered heatmap of EOS and control specimen values for the 5 differentially abundant proteins. For **B**, missing protein abundance values were imputed. (**C**) Distribution of protein abundance in EOS and control specimens for each protein. Box plots show median and interquartile range. Whiskers extend to the last point within 1.5× interquartile range of the box. Bee swarms show individual samples. Comparisons were significant by Mann-Whitney *U* test with Benjamini-Hochberg FDR adjustment.

Principal component analysis (PCA) of the protein abundance data for CRP, LBP, SAA1, LRG1, and SERPIN3A resulted in a clear separation of most of the EOS and control specimens, with axes accounting for 84% of the variance (Figure 2A). Hierarchical clustering by the abundance of these 5 proteins likewise shows a clear EOS cluster, though with a few control specimens interspersed (Figure 2B). Protein abundance for all 5 proteins was significantly different between the EOS and control specimens (Figure 2C). Results were consistent, showing significantly different protein abundance when analysis included or excluded full-term GA infants (Supplemental Figure 2B). The distributions for CRP overlapped more than the other proteins. These data suggest that the measurement of one or more of these putative biomarkers in cord blood might be sufficient to differentiate healthy infants from those with EOS.

### Quantitative immunoassays confirm elevated CRP, SAA, and LBP in EOS cord blood specimens.

**Figure 3:**
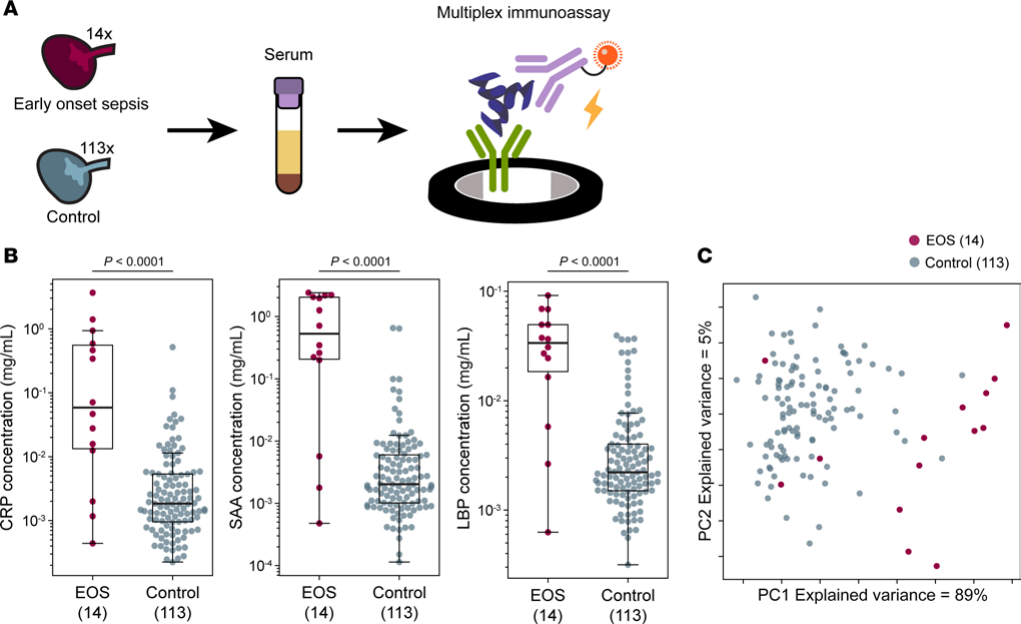
Quantitative multiplex immunoassay detection of potential EOS biomarkers. (**A**) Diagram of experimental procedure. (**B**) Distribution of protein concentration in mg/mL in EOS and control specimens for each protein. Box plots show median and interquartile range. Whiskers extend to the last point within 1.5× interquartile range of the box. Bee swarms show individual samples. Comparisons were significant by Mann-Whitney *U* test with Benjamini-Hochberg FDR adjustment. (**C**) PCA of EOS and control specimen protein abundance based on MSD data.

Though useful for target discovery, shotgun proteomic data cannot be used to rule out the presence of a protein in a specimen, nor is it a clinically feasible approach. Therefore, we next sought to validate these findings using commercially available, quantitative immunoassay kits to detect CRP, SAA1/SAA2, and LBP (Figure 3A). Only preterm controls (*n* = 105) were included for better comparison to our infants with EOS (all <37 weeks). Once again, the concentrations of the 3 biomarkers were significantly elevated in the EOS compared with the control specimens, though for each biomarker 3 EOS specimens had lower concentrations more comparable to the control samples (Figure 3B and Supplemental Figure 3). PCA of these data demonstrated that though most of the EOS specimens formed a distinct cluster, 3 EOS specimens clustered with the control specimens (Figure 3C). These 3 infants had different GAs at birth (24 5/7, 33 5/7, and 34 3/7), and blood cultures yielded typical septic pathogens (*E*. *coli*, *E*. *coli*, and *K*. *oxytoca*, respectively) (Table 2). Notably, however, these 3 infants had the longest intervals between birth and a positive blood culture (drawn at 65, 55, and 62 hours of life, respectively). The two infants with *E*. *coli* had a negative blood culture in the first day of life, whereas the infant with *K*. *oxytoca* developed emesis and increased abdominal girth on day 3 of life with an abdominal radiograph concerning for pneumatosis, prompting a septic workup. The other 11 infants with EOS had a positive blood culture that was drawn within the first few hours of life.

### Predictive modeling of EOS.

To assess the predictive value of these putative biomarkers versus currently referenced clinical variables, we made a series of small logistic regression models containing sex, GA, and either a single biomarker or single clinical variable utilizing immunoassay results (Table 3). Clinical variables analyzed included plasma protein concentration, multiple gestations, chorioamnionitis, preeclampsia, PROM, route of delivery, labor, and a delivery sum score factoring in both the delivery route and if the mother went into labor. To improve the predictive power for in utero–derived EOS, the 3 outlier infants were excluded from modeling. The logistic model containing a biomarker had the lowest Akaike information criteria (AIC) than those containing a clinical variable, with the best-performing single clinical variable being the presence of clinical chorioamnionitis. We next explored multivariate logistic regression models. Most clinical variables as operationalized have a small cardinality and are colinear with at least one other variable, posing a challenge for meaningful model selection and interpretation of variable importance; we thus selected single clinical variables from correlated variable sets and evaluated model performance of these pruned formulae (Supplemental Figure 4 and Table 3). Model performance for the best performing set of clinical variables with increasing representation of biomarkers (none, SAA only, or all 3) showed both improved classification performance and reduced AIC with the addition of SAA. However, the best performing models had recall of 0.82, indicating that a substantial fraction of authentic EOS cases would be missed were these models used for prediction.

**Figure 4:**
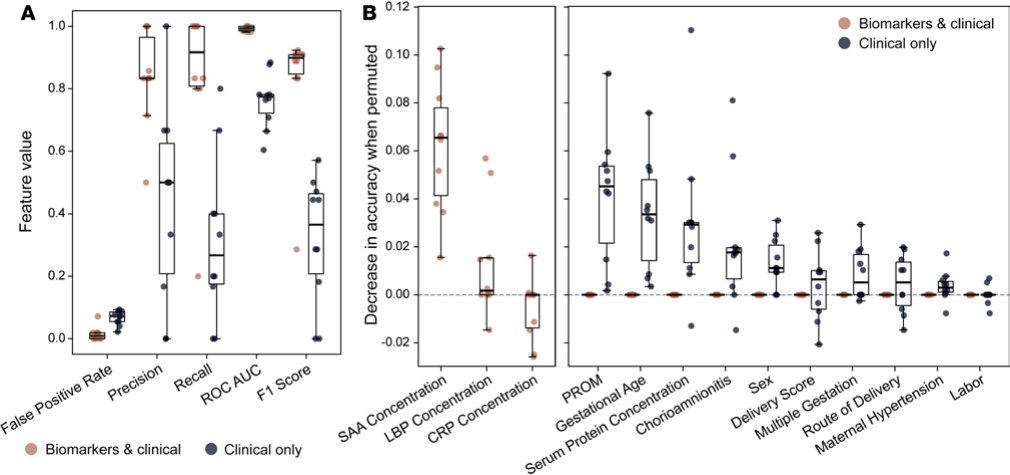
Modeling of EOS risk using biomarkers. (**A**) Model fit parameters for random forests models trained with (amber) or without (black) cord blood biomarker concentrations as a factor. Metrics are calculated with EOS as the positive class. Points represent performance for a single run of the model. Box plots show the median and interquartile range. Whiskers extend to the last point within the 1.5× interquartile range of the box. (**B**) Permutation variable importance for variables in the random forest model with cord blood biomarker concentrations included. Biomarker concentrations are shown on the left. Variables included in both models are shown on the right. Box plots show median and interquartile range. Whiskers extend to the last point within 1.5× interquartile range of the box. Points represent performance for a single run of the model.

To employ a modeling strategy more robust to the small sample size and variable selection challenges of our dataset, we produced random forest (RF) models with or without the biomarker concentrations. As with the logistic models, RF models were built initially with and subsequently without the 3 outlier infants identified in PCA analysis (Figure 3 and Table 2) to focus on diagnosis of EOS present at birth. Random forest models that included the 3 biomarker concentrations outperformed models with only clinical variables in precision, recall, receiver operating characteristics, and F1 score (Figure 4A). Importantly, addition of these biomarker data improved recall, consistent with the clinical importance of early identification of every EOS case, and had greater precision, consistent with the clinical goal of minimizing unnecessary antibiotic treatment in infants who will not go on to develop sepsis. However, recall remained below 100%, indicating that complete sepsis identification requires strategies beyond this set of clinical variables and cord blood biomarkers.

To further characterize the value of cord blood biomarker levels, we measured variable importance by permutation in both RF models (Figure 4B). In this strategy, the values of individual variables are randomly shuffled as the model is applied to multiple data splits. Degradation of model performance is thus interpretable as the fractional contribution of each variable to model accuracy. For the RF model with both biomarkers and clinical variables, SAA concentration had the greatest contribution to model performance by far, highlighting the value of this biomarker, followed by LBP concentration. None of the clinical variables contributed to accuracy of the biomarker-inclusive RF model by this metric. For the RF model with only clinical variables, PROM, GA, serum protein concentration, and chorioamnionitis were the most important variables, consistent with known EOS risk factors. That the biomarker-added model had improved performance while relying almost solely on biomarker concentrations suggests that these biomarkers have predictive value beyond clinical variables alone. This RF model also performed better than the best multifeatured logistic regression models with fewer false negatives, which is important given the clinical importance of negative predictive value and risk of missing true EOS (Table 3, Recall). Together, these data suggest that cord blood biomarkers may be useful adjuncts to clinical risk factors in EOS management.

### Assessment of biomarkers in infants with PS.

**Figure 5:**
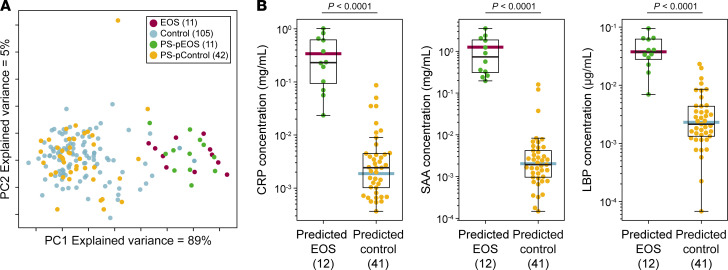
Presumed sepsis cases categorized by random forest model. Biomarker concentrations in cord blood from presumed sepsis cases were measured by immunoassay; cases were then categorized as either predicted EOS (PS-pEOS) or predicted control (PS-pControl). (**A**) PCA plot of biomarker concentrations in cases colored by status and predicted status. (**B**) Concentration of biomarker proteins in predicted EOS and predicted control cases. Box plots show median and interquartile range. Whiskers extend to the last point within the 1.5× interquartile range of the box. Points represent individual samples. Wide red lines show the median value for ascertained EOS cases (excluding outliers); wide blue lines show the median value for ascertained controls. Comparisons were significant by Mann-Whitney *U* test with Benjamini-Hochberg FDR adjustment.

Most infants receiving antibiotics early in life do not have positive blood cultures and so are treated as PS. Infants with PS presumably include a mixture of infants with and without infection. In proteomics analysis, they did not cluster distinctly from EOS and control group infants (Supplemental Figure 5). To assess the outcome of our model in predicting sepsis in this population, we performed the same immunoassays on cord blood specimens from infants with PS. Of the 53 infants with PS, our machine learning model predicted 41 to resemble the healthy infants in the control group, and 12 were predicted to resemble the infants with EOS. PCA of the immunoassay data confirmed a clear clustering of the 12 infants with PS predicted to have EOS with the infants who had true EOS, while the other 41 clustered cleanly with the control group (Figure 5A). The infants with PS predicted to have EOS had significantly higher levels of CRP, SAA1, and LBP than the infants predicted to be in the control group, with median values near or exceedingly those of the infants with true EOS (Figure 5B). That the majority of the infants with PS would potentially not have sepsis would be consistent with the risk averse paradigm of prolonged empiric antibiotic administration upon clinical suspicion of sepsis.

## Discussion

In this study, we used an unbiased proteomics discovery strategy to identify candidate EOS biomarkers in cord blood at the time of birth, 3 of which were independently verified by immunoassay and proved useful as predictors in a random forest classifier. Our final random forest model included all 3 biomarkers along with clinical features, and the performance was superior to our logistic regression models. The random forest classifier has a high negative predictive value, which could be used to rule out EOS and help clinicians better target antibiotic treatments to those infants with high likelihood of culture-positive EOS. Negative findings on cord blood screening for these biomarkers could increase clinical justification for withholding of empiric antibiotics after birth among low-risk infants or early discontinuation among higher-concern infants with negative blood culture results at 1–2 days of life, respectively. In other words, this diagnostic could be sent from cord blood at birth for all preterm infants with risk factors for infection or those born to mothers with clinical chorioamnionitis. Infants may be started on antibiotics empirically or not, but with cord blood marker results available within hours, these data can be utilized to both ensure infants with EOS are receiving antibiotics and to inform cessation of antibiotics with confidence for infants with low-risk cord blood biomarker profiles at the time that a blood culture result is negative. This could reduce unnecessary prolonged antibiotics exposure among neonates by adding more objective data to the diagnosis and management strategy of EOS.

Our sample of 14 culture-confirmed EOS cases and GA-matched individuals acting as controls and PS cases is larger than has previously been used for similar studies, providing power to detect differences in proteomic data. Additionally, our precise categorization of culture-proven EOS versus PS cases further strengthens inferential power by removal of clinically diagnosed but uncertain cases of culture-negative sepsis, which may otherwise dilute candidate biomarker differences between EOS and controls . Importantly, both control and EOS arms of our dataset were enriched in preterm infants, who are most susceptible to EOS, most vulnerable to EOS morbidity, and most vulnerable to adverse consequences of prolonged early antibiotics exposure (2, 3). The most widely used EOS screening tool, the Kaiser Permanente Sepsis calculator, is not applicable for infants under 34 weeks’ gestation. Thus, the biomarkers we have identified here meet a significant unmet need for improved EOS screening in the preterm population. Only 22% (12 of 53) of infants with PS treated for EOS had biomarker levels consistent with culture-proven EOS cases. While further validation and observational studies are necessary, these results illustrate the potential for a noninvasive test at birth to limit antibiotic exposure to as many as 78% of infants who receive a prolonged empiric treatment course in the setting of negative blood culture.

We identified 5 candidate biomarkers of EOS in umbilical cord blood: SAA1, LBP, CRP, LRG1, and SERPINA3. All 5 are known to be acute-phase reactants. The presence of these proteins in cord blood is consistent with known EOS pathology. LBP is a plausible marker because it is involved in the acute-phase immunologic response to Gram-negative bacterial infections, a common EOS cause. CRP and SAA1/2 are produced by hepatocytes upon IL-6 and IL-1–like cytokine stimulation early in the acute-phase response. CRP is widely used in clinical practice, although it is not specific for inflammation of infectious origin and is not routinely measured in cord blood. We previously identified CRP and SAA as potential biomarkers of EOS in a targeted screen of acute-phase reactant proteins in cord blood (24). Our unbiased proteomic workflow identified an overlapping set of proteins, strengthening the notion that these acute-phase reactants may be useful cord blood biomarkers of EOS. An independent serum proteome analysis of sepsis in children previously identified an overlapping panel of biomarkers: SAA1, LRG1, and sCD25 (30). Several other studies have also pointed to acute-phase reactants, including SAA1, as sepsis biomarkers in multiple populations, constituting a consensus that these proteins may be useful for sepsis diagnosis (31–34). Here, we extend these findings to the cord blood of a uniquely vulnerable population of premature infants for whom postnatal markers vary due to birth and other noninfectious comorbidities.

An important caveat to using this candidate set of cord blood biomarkers for EOS diagnosis is that it only identifies EOS developed in utero. Cord blood markers can only provide information about the inflammatory state of the infant at the time of delivery, until clamping of the umbilical cord. Although this makes cord blood a valuable source of information about the intrauterine environment where EOS typically develops, it also makes cord blood uninformative for the subset of neonatal sepsis that develops later within the defined 72-hour window for EOS but is not present at the time of birth (i.e., blood culture at birth is negative and infection/necrotizing enterocolitis developed postnatally). Indeed, for the 3 culture-confirmed EOS cases with low cord blood biomarkers, culture positivity was only observed after a second blood culture was requested after 48 hours of worsening clinical status. If cord blood biomarker screening were adopted, ongoing monitoring of signs, use of clinical judgment, and combination with existing microbiologic tools would remain key for EOS management.

Additional limitations of the study include the small number of confirmed EOS cases, which can complicate machine learning models due to a risk of overfitting. While we employed several orthogonal approaches to measure these biomarkers and model these data, follow-up studies with independent cohorts across multiple sites will be required to add robustness to the data.

Additional prospective work is necessary to determine whether stratification of EOS risk and antibiotic treatment based on inflammatory biomarker screening of cord blood improves the balance of EOS avoidance and antibiotic stewardship. In both our Northwestern University Cord Blood biorepository (NU Cord) cohort and national studies, the percentage of very low-birth-weight infants receiving prolonged antibiotic courses (~26%) (9) far outweighs those with culture-confirmed infection (~1%) (3). Though not all authentic sepsis cases may be confirmed by culture owing to pathogen fastidiousness, low sample volume, and maternal antibiotics, this likely reflects significant overtreatment. Reducing the duration of exposure to antibiotics could reduce the harms of overtreatment. The logistics and efficacy of cord blood biomarker screening require further validation in subsequent, multicenter studies.

## Methods

### Sex as a biological variable.

Male and female neonates were both included in the study. Modeling and analysis included both sexes.

### Ethics, enrollment, and participant selection.

We utilized archived cord blood plasma from an ongoing prospective study of infants born at Northwestern Prentice Women’s Hospital between 2008 and 2019 (NU Cord). To identify potential cord blood biomarkers of EOS, we selected 3 cohorts of neonates with banked cord blood: 14 who were treated for microbially confirmed EOS, 56 who were treated for EOS with negative culture results (PS), and 150 control infants with no suspicion of sepsis (cohort previously described in ref. 29). Samples in this investigation were selected based on proven EOS (defined as positive blood culture with true pathogen [i.e., not a coagulase-negative staphylococcal species, corynebacterium, or other nonpathogenic species] within 72 hours of life, ref. 16; clinical sepsis defined as instability in respiratory, hemodynamic, and/or end-organ dysfunction along with abnormal laboratory markers; and antimicrobial treatment ≥7 days, *n* = 14). Individuals in the control group were selected based on GA and the absence of presumed or proven EOS (i.e., the infant received no antibiotic treatment course for sepsis within the first 72 hours of life and had no positive microbiologic sterile site cultures). A total of 150 infants were frequency matched within each GA category (epochs: 25–28 weeks, 29–32 weeks, 33–36 weeks, 37–42 weeks) ± 2 weeks to the best of our ability within the cohort. There were approximately equal numbers by sex and route of delivery (vaginal delivery vs. cesarean delivery with or without labor). A third group of cord blood was identified from infants with PS, where infant had symptomatic clinical illness and no positive cultures (blood, cerebrospinal fluid, or respiratory), yet received a ≥7-day antibiotic treatment course for culture-negative presumed EOS due to instability starting in first 72 hours of life (*n* = 56). Infants with a positive culture soon after 72 hours (i.e., within the first week of life) or with congenital abnormalities, such as cardiac anomalies or diaphragmatic hernia, were excluded from this analysis. Infants with PS were frequency matched within the EOS cohort by GA (±2 weeks), route of delivery, and sex (Table 1).

### Blood and data collection.

Venous cord blood was collected in purple/lavender top EDTA tubes and refrigerated until centrifugation at 700 g for 10 minutes. Time from collection to centrifugation varied from 0 to 4 days. Plasma was separated from red blood cell and buffy coat into aliquots stored at –80°C until use. Clinical data, including birth weight, comorbid conditions, antenatal corticosteroid administration, and delivery characteristics, were collected from the electronic medical record.

### Proteomics sample preparation.

Banked cord blood samples were thawed and processed for proteomics as previously described (29). Briefly, 14 highly abundant proteins were depleted (Top 14 Abundant Protein Depletion Spin Columns; Thermo Scientific) from plasma equivalent to 600 μg protein. Proteins were then precipitated, reduced, alkylated, and digested with trypsin. The resulting peptides were desalted on C18 columns, eluted in acetonitrile and formic acid, and reconstituted in 0.1% formic acid aqueous solution. Peptides were then analyzed by LC-MS/MS on an UltiMate 3000 Rapid Separation nanoLC coupled to an Orbitrap Elite mass spectrometer (both from Thermo Fisher Scientific) with data-dependent acquisition of the top 15 precursors. Samples were run in duplicate across 4 batches with pooled controls to assess batch-to-batch variance. Batches were randomized with a stratified sampling approach to balance distribution of GA, sex, and EOS.

### Proteomics data analysis and normalization.

Peptides were identified against the SwissProt human database using the Andromeda search engine in MaxQuant (version 1.6.0.16) with FDR <1% for protein identification. LFQ was conducted using MaxLFQ. Log_2_-transformed LFQ values were used for all statistical analyses. To correct for batch effects, batch normalization was performed as previously described (29). In summary, proteins detected in only one batch were excluded, and proteins detected in multiple batches were normalized by subtraction of the mean log_2_ LFQ protein abundance difference in the pooled control samples between the nth batch and the first batch. Batch-normalized protein abundance was then averaged across technical replicates of each sample.

### Immunoassay biomarker detection.

Quantitative biomarker detection was performed using Meso Scale Discovery (MSD) immunoassay kits: Vascular Injury Panel 2, which measures SAA and CRP (MSD, K151A9H), and an R-PLEX singleplex detection assay for LBP (MSD, K151K5R). Assays were performed according to the manufacturer’s instructions, in technical duplicate, from 10 μL cord blood plasma from a subset of the same samples utilized for proteomics (*n* = 14 EOS, *n* = 105 controls, and *n* = 53 PS). The infants in the control group included in this validation cohort were exclusively preterm infants (GA range 25 6/7 to 36 6/7 weeks). Immunoassays were performed in Northwestern University’s Immunotherapy Assessment Core.

### Statistics.

For pairwise comparisons between nonnormally distributed populations, significance was assessed by Mann-Whitney *U* test with Benjamini-Hochberg FDR correction. Protein abundance was visualized using hierarchically clustered heatmaps, which were produced using the *clustermap* function of *seaborn* (v 0.13.2). To enable heatmap visualizations, missing protein values were imputed using a 2-step approach applied separately for each sample type. For proteins with <30% missingness, values were imputed using an interative imputer (*scikit-learn* v 1.5.1 IterativeImputer). For proteins with greater missingness, values were imputed by random sampling from the lowest decile of observed values for each protein. All statistical analyses were conducted on the original, nonimputed dataset.

PCA and random forest modeling were performed using *scikit-learn* (v 1.5.1). For logistic regression metrics, we used the default 0.5 probability threshold for classification. Model selection was informed by AIC. For the random forest classifier, 10 runs using a stratified shuffle split allocation of 50% test and 50% training data were performed. The training data were further subjected to gridsearch cross validation. EOS samples were assigned 10:1 class weight to mitigate class imbalance. Hyperparameter tuning explored combinations of n-estimators 50, 100, splitting criteria (Gini, entropy), max tree depth 4–6, and minimum samples per leaf 2–3. Models consisting solely of clinical variables (GA, chorioamnionitis, PROM, etc.) as well as biomarkers in conjunction with clinical variables were explored. Variable importance was assessed by permutation importance with 20 repetitions. For the demographics table (Table 1), differences in continuous nonnormally distributed variables were assessed by Mann-Whitney *U* test, while differences in distribution of categorical variables were assessed by χ^2^ test. All analyses were performed in *python* (v 3.12.7). *P* values of less than 0.05 were considered significant.

### Study approval.

This study was approved by the Institutional Review Boards of Northwestern University (STU00201858) and Lurie Children’s Hospital (IRB 2018-2145). Parental informed consent was obtained for use of clinical data and infant cord blood samples. All research activities were performed in accordance with the Declaration of Helsinki.

### Data availability.

Proteomics data have been deposited to the ProteomeXchange Consortium via the PRIDE partner repository with the dataset identifier PXD051974. Python code used for analysis is available at https://github.com/markebecker/eos_biomarkers (branch: main, commit ID: d28a56a381bcd533f0c36fb6695c99a4ce7c16ef).

## Author contributions

LBM provided conceptualization, methodology, data curation, analysis, funding acquisition, writing of the original draft, and review and editing of the manuscript. MEB, TLH, and JFH provided methodology, data curation, formal analysis, visualization, writing of the original draft, and review and editing of the manuscript. YG provided methodology, investigation, data curation, and review and editing of the manuscript. DS, NL, AK, SP, Y Li, Y Luo, and SO provided methodology, investigation, and review and editing of the manuscript. WG provided conceptualization, methodology, supervision, and review and editing of the manuscript. KKM provided conceptualization, methodology, data curation, supervision, and review and editing of the manuscript. PCS provided conceptualization, methodology, data curation, supervision, review and editing of the manuscript, and funding acquisition. All authors contributed to the article and approved the submitted version.

## Supplementary Material

Supplemental data

ICMJE disclosure forms

Supporting data values

## Figures and Tables

**Table 1:**
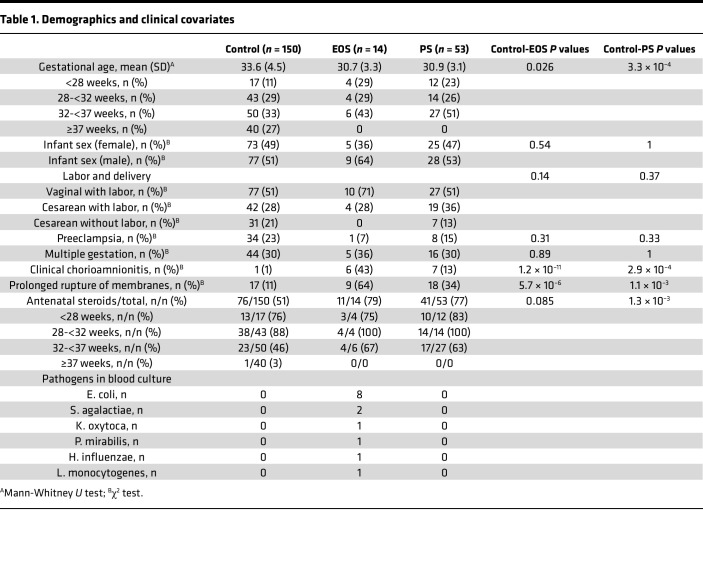
Demographics and clinical covariates

**Table 2:**
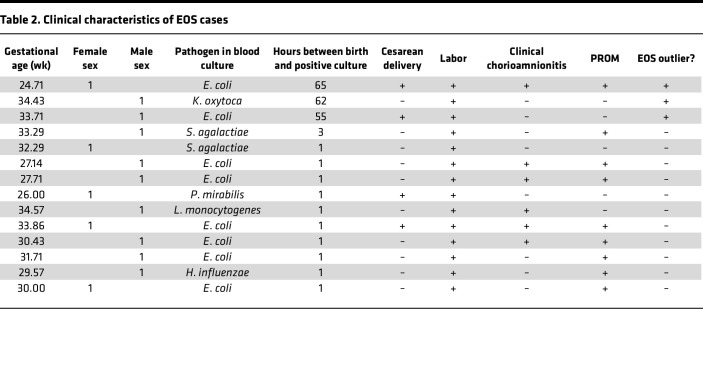
Clinical characteristics of EOS cases

**Table 3:**
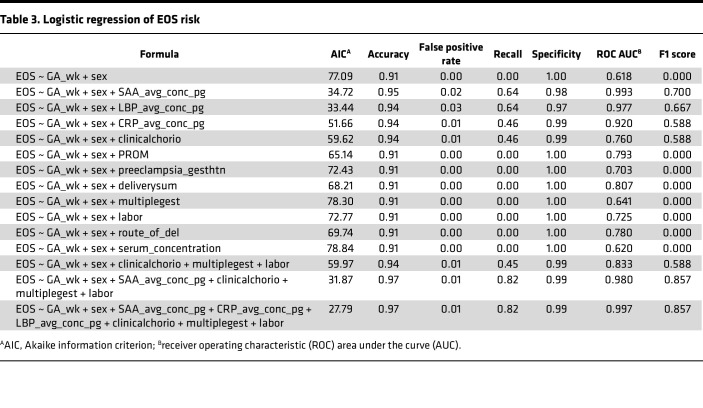
Logistic regression of EOS risk

## Version 1 06/10/2025

In-Press Preview

## Version 2 07/08/2025

Electronic publication
