# Supplementary material for: Cord blood proteomics identifies biomarkers of early-onset neonatal sepsis
Source: JCI Insight. 2025 Jun 10;10(13):e193826. doi: 10.1172/jci.insight.193826 (PMC12288901; doi:10.1172/jci.insight.193826)
Supplement: Supplemental data [file jciinsight-10-193826-s220.pdf]

Supplemental Material

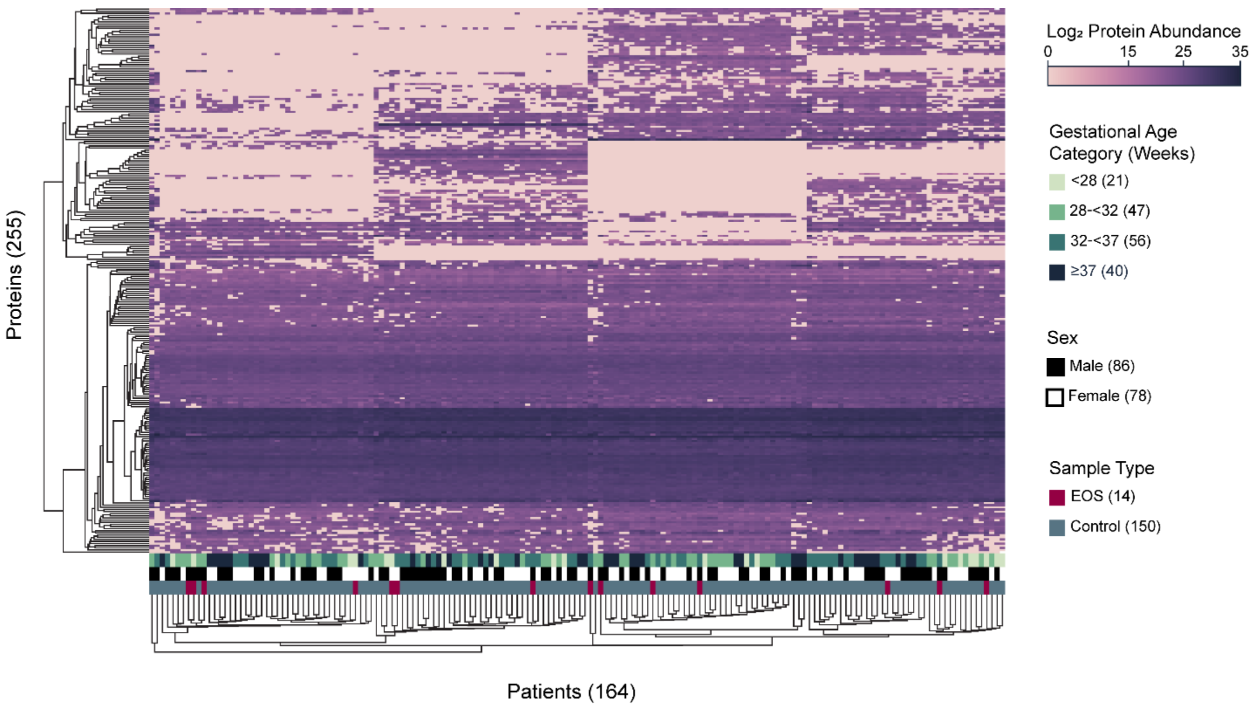

Supplemental Figure 1 | Non-imputed heat map of protein abundance. Hierarchically clustered heat map of protein abundance in cord blood for EOS and control specimens. Proteins with missing mass spectrometry data were imputed with zeroes. Specimens are clustered by gestational age category, sex, and sample type.

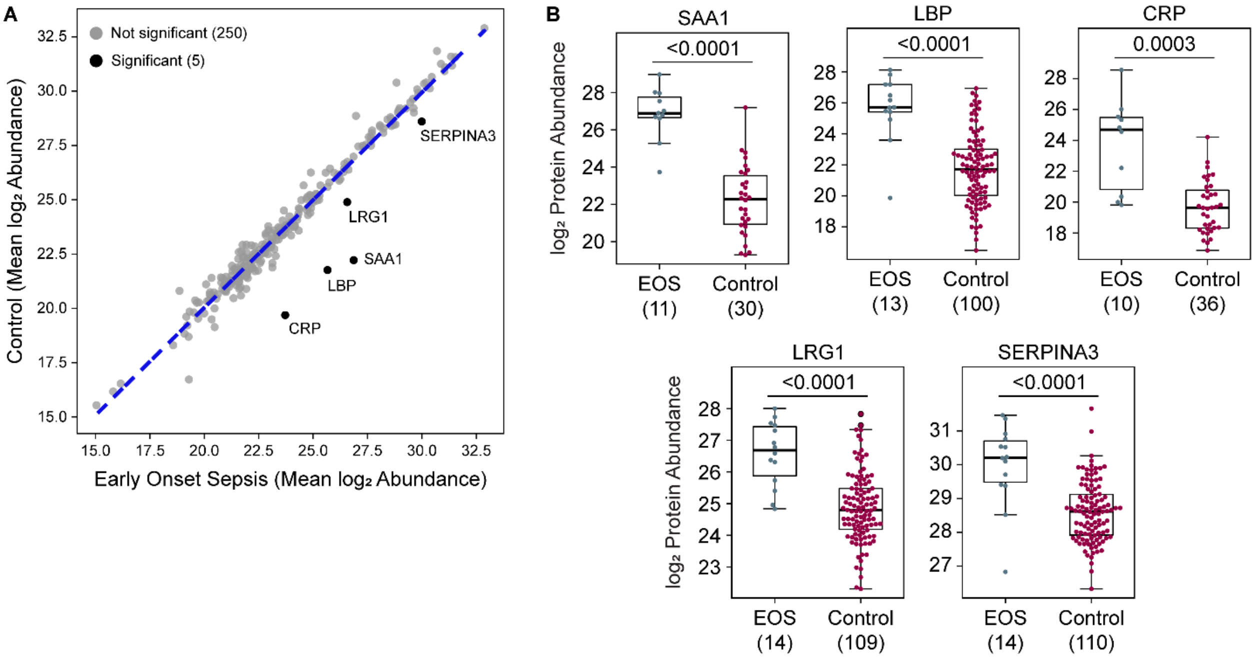

Supplemental Figure 2 | Differential protein abundance among pre-term infants only (gestational age below 37 weeks). A, Plot of mean abundance of proteins in EOS and control specimens. Black points were significant by Mann-Whitney U test with Benjamini-Hochberg false discovery rate adjustment ($p < 0.05$). **B,** Distribution of protein abundance in EOS and control specimens for each protein. Box plots show median and interquartile range. Whiskers extend to the last point within 1.5x interquartile range of the box. Bee swarms show individual samples. Comparisons were significant by Mann-Whitney U test with Benjamini-Hochberg false discovery rate adjustment.

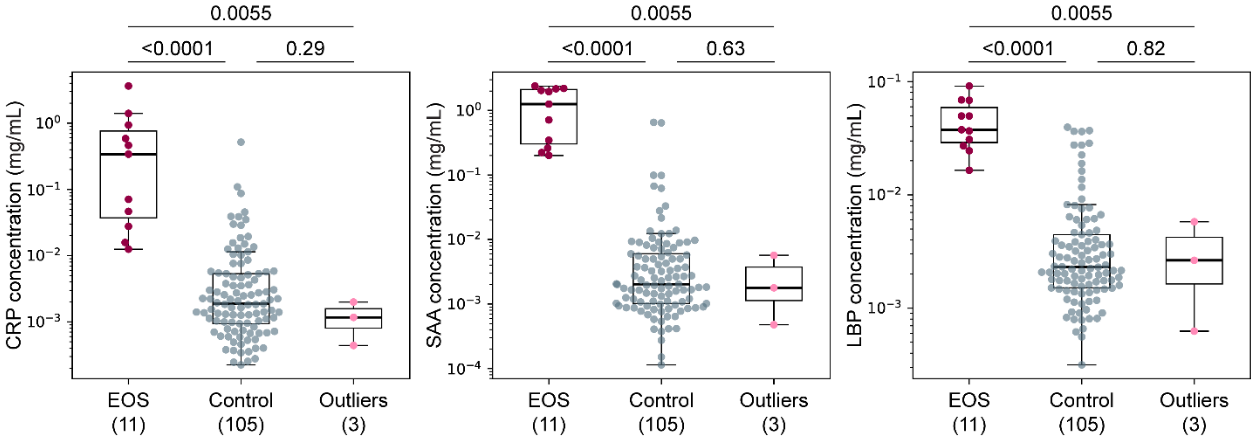

Supplemental Figure 3 | Quantitation of potential EOS biomarkers with EOS outliers separated. Distribution of protein concentration in mg/mL in EOS and control specimens for each protein. Box plots show median and interquartile range. Whiskers extend to the last point within 1.5x interquartile range of the box. Bee swarms show individual samples. Comparisons were significant by Mann-Whitney U test.

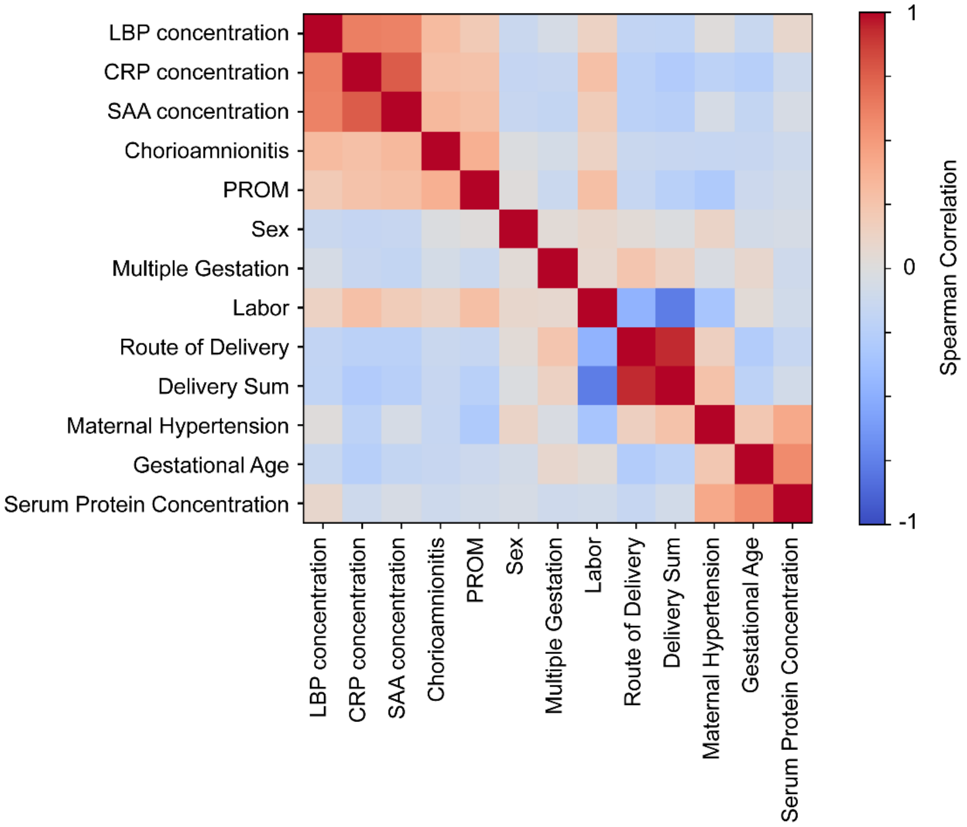

Supplemental Figure 4 | Correlation of variables. Heat map of Spearman's rho of variables used for modelling of EOS risk.

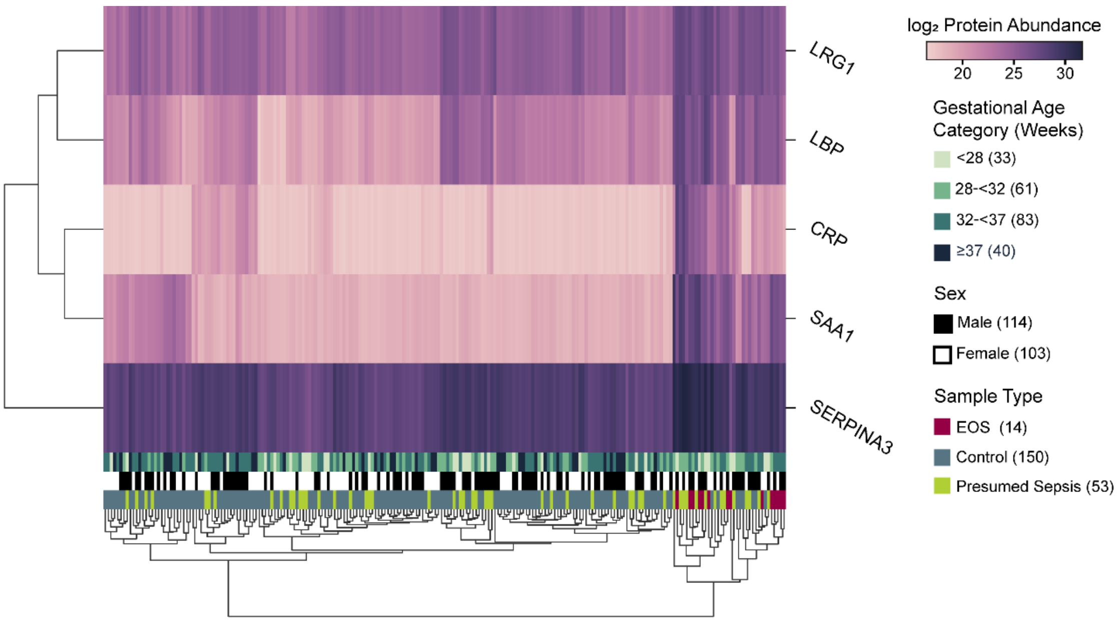

Supplemental Figure 5 | Differentially abundant proteins by mass spectrometry in presumed sepsis group. Clustered heatmap of EOS, control, and presumed sepsis specimen values for the five differentially abundant proteins. Missing protein abundance values were imputed.
